# Our 12th Voyage

**Published:** 1896-07

**Authors:** 


### THE JOURNAL'S OWN PAGE.

**Boom! Boom!** How the "Red Back" booms:

"I want to say, your June number contains more good, solid, logical food than I have lately seen within so small a compass."

SAM R. BURROUGHS, M. D.

**The** June number of the "Red Back" received; and while eagerly devouring its contents, I came across your request to physicians to send you the name and address of all persons practicing by virtue of Texas Health College diplomas. * * * May the TEXAS MEDICAL JOURNAL grow in greatness and usefulness, as it deserves, and the profession of medicine will continue to have a friend who will watch and protect their every interest. With kindest regards,

T. P. DAVIS, M. D., Canton, Texas.

**Another.**—Dr. J. H. Evans, the physician to the State Penitentiary at Huntsville, in sending $4 for two years subscription, says: "I can not get along without the 'Red Back,' money or no money." (But the doctor always sends the money.)

OUR TWELFTH VOYAGE.—(Excuse us while we do a little crowing.) Eleven years ago, 10th July, 1885, the TEXAS MEDICAL JOURNAL,—then "Daniel's Texas Medical Journal,"—a little unpretentious 5x8 pamphlet of 48 pages, all told, made its first venture. It was a timid venture; but it told. It met with an almost unhoped for reception at the hands of the best men of the Texas medical profession, who cordially gave it their support, moral and financial. Thus encouraged, it developed a plucky spirit; buckled on its armor and went into the fight against quackery of every kind, in and out of the profession, making *organization* of the rank and file of the profession its key-note, and advocating, first, last, and all the time, a higher standard of medical education and morals. It has devoted its best energies, for these eleven years, to the accomplishment of these aims—meantime growing in strength as it grew in years and patronage, till now, it is conceded to be the *foremost medical journal* in all the Southwest, including Texas, Louisiana, Arkansas and Oklahoma (see statement to that effect, voluntary, from the American Newspaper Directory), and its circulation rating is the highest of any similar publication in that territory.

The JOURNAL is now a recognized power,—a valuable property which money couldn't buy,—and its influence is felt and acknowledged throughout the United States, it being oftener quoted or referred to, it is said, by contemporaries, than any journal published. It has an individuality; it is *sui generis;* it wages war, as the schoolmaster said, upon the individuality of its own personal curve; i. e., in the vernacular—its own hook. The policy—heretofore—"free lance," its motto, "see a (quack's) head and hit it," have cost its owner and engineer—yours, truly—a good deal of money—in one way and another, which it is unnecessary to mention (we have "piped," while others danced, and, of course, have made some—many—enemies.) But it must be a very poor-spirited creature, indeed, who could go through this world—a poor journal, indeed, that could go into and through the controversies which the *Red Back* has engaged in, without making enemies. It would be a poor compliment to any man to say, "he has not an enemy." We love to have some men for enemies; we love to hate them (but oh, how much better we love to love our friends).

In short, resting on our oars, and taking a short breathing spell, after the completion of our eleventh voyage, we have indulged in the above retrospect; now to the future.

The demand for the JOURNAL has been such as to compel the management—very reluctantly—to cut off about one-half its exchanges; and necessitate the addition of twenty per cent. to its regular monthly issue (since the publication of the statement by the American Newspaper Directory above referred to). Everybody has wanted to see the Red Back, that they have heard so much talk about. We want to spread ourselves still more—and with this increased circulation, we are going to reach out for new territory. The circulation of the JOURNAL is *bona fide*. Of course, we send out some sample copies, but not as a rule, and our circulation is not a sample copy circulation.

We want, and hereby solicit, the influence and co-operation of every reputable physician who reads this. The JOURNAL is engaged in a mission almost holy; and in the fight against humbuggery, and the demand for a higher standard of education and professional character, it should have the cordial support of all good men. We ask our friends—our old, tried and true supporters, who even in the darkest hour never once flickered in their allegiance to the little Red Back, the exponent of rational medicine and ethical deportment—to talk some for us. Show your copy to "some forlorn brother," ship-wrecked or not, on whose dark mind the light of the Red Back has not before fallen; and tell him what a good thing it is; how much solid comfort and useful information you have gotten out of it, and how many chuckles you have had at some of its "get-offs"; in brief—get his subscription.

All aboard now for the twelfth trip. We set sail with a stout heart, and a strong hope, and with a determination not the least diminished, to get there; to get where we started. Having made the JOURNAL what it is, to keep it at the top notch of excellence, and to give to our readers the *best two dollars' worth* (in monthly installment) they ever had in the way of a medical journal. So long!

